# The Relationship of Kidney Function, Cardiovascular Morbidity, and All-Cause Mortality: a Prospective Primary Care Cohort Study

**DOI:** 10.1007/s11606-022-07885-8

**Published:** 2022-12-21

**Authors:** Päivi E. Korhonen, Sem Kiiski, Hannu Kautiainen, Seppo Ojanen, Risto Tertti

**Affiliations:** 1 Department of General Practice, Turku University and Turku University Hospital, Turku, Finland; 2 Folkhälsan Research Center, Helsinki, Finland; 3 Unit of Primary Health Care, Kuopio University Hospital, Kuopio, Finland; 4 Department of General Practice and Primary Health Care, University of Helsinki and Helsinki University Hospital, Helsinki, Finland; 5 Department of Internal Medicine, Tampere University Hospital, Tampere, Finland; 6 Department of Internal Medicine, Vaasa Central Hospital, Vaasa, Finland; 7 Department of Internal Medicine, Turku University, Turku, Finland

**Keywords:** glomerular filtration rate, renal hyperfiltration, mortality, cardiovascular diseases

## Abstract

**Background:**

Lower-than-normal estimated glomerular filtration rate (eGFR) is associated with the risk for all-cause mortality and adverse cardiovascular events. In this regard, the role of higher-than-normal eGFR is still controversial.

**Objective:**

Investigate long-term clinical consequences across the levels of eGFR calculated by the creatinine-based Chronic Kidney Disease Epidemiology Collaboration (CKD-EPI) equation among apparently healthy cardiovascular risk subjects.

**Design:**

Prospective study.

**Participants:**

Participants (*n*=1747) of a population-based screening and intervention program for cardiovascular risk factors in Finland during the years 2005–2007.

**Main Measures:**

Cardiovascular morbidity and all-cause mortality.

**Key Results:**

Over the 14-year follow-up, subjects with eGFR ≥105 ml/min/1.73 m^2^ (*n*=97) had an increased risk for all-cause mortality [HR 2.15 (95% CI: 1.24–3.73)], incident peripheral artery disease [HR 2.62 (95% CI: 1.00–6.94)], and atrial fibrillation/flutter [HR 2.10 (95% CI: 1.21–3.65)] when compared to eGFR category 90–104 ml/min after adjustment for cardiovascular and lifestyle-related risk factors. The eGFR category ≥105 ml/min was also associated with a two-fold increased mortality rate compared to the Finnish general population.

**Conclusions:**

Renal hyperfiltration defined as eGFR ≥105 ml/min/1.73 m^2^ is a frequent and important finding in patients commonly treated in primary care. These patients should be followed closely for timely interventions, such as strict BP and blood glucose regulation.

**Supplementary Information:**

The online version contains supplementary material available at 10.1007/s11606-022-07885-8.

## INTRODUCTION

In general population cohorts, a reverse J-shaped association between all-cause mortality and estimated glomerular filtration rate (eGFR) values has been reported.^1^ Lower than normal eGFR is associated with the risk for all-cause mortality, cardiovascular disease (CVD) events, arrhythmias, and progression of heart failure, and it may further develop into end-stage kidney disease.^2–5^ It is estimated that chronic kidney disease (CKD) will be the fifth leading cause of death globally by 2040.^6^ Higher than normal eGFR, i.e., renal hyperfiltration, has also been suggested to predict mortality, CVD, diabetes, and CKD.^5, 7–8^ However, the association of renal hyperfiltration and adverse events is still controversial due to study differences in the definition of hyperfiltration, methods to estimate GFR, variations in co-morbidities, and other confounding factors such as muscle mass.^7^ Thus, the current guidelines recommend screening for only CKD among high-risk subjects, e.g., hypertensives, diabetics, and those with family history of chronic kidney diseases.^9^

Early stages of CKD are often asymptomatic or have non-specific symptoms. Therefore, CKD usually remains undiagnosed for a long period until chance findings from screening tests appear or when symptoms become severe.^10^ Conventional risk factors for CKD are high blood pressure (BP), glucose disorders, and high body mass index (BMI).^11^ Development and complications of CKD, such as CVD, may well be prevented by addressing these risk factors and adverse lifestyle factors.^12^ This said, primary health care has a major role in early diagnosis and slowing the loss of kidney function.

A population-based screening and intervention program for CVD risk factors and diabetes was conducted in a primary health care setting in Finland during the years 2005–2007.^13^ We report herein the findings on cardiovascular morbidity and all-cause mortality after 14 years of follow-up. Specifically, our aim was to investigate if the long-term clinical consequences varied between the levels of eGFR at baseline, and to compare mortality between the study participants and the general population of Finland over the same period of time.

## METHODS

**Figure 1:**
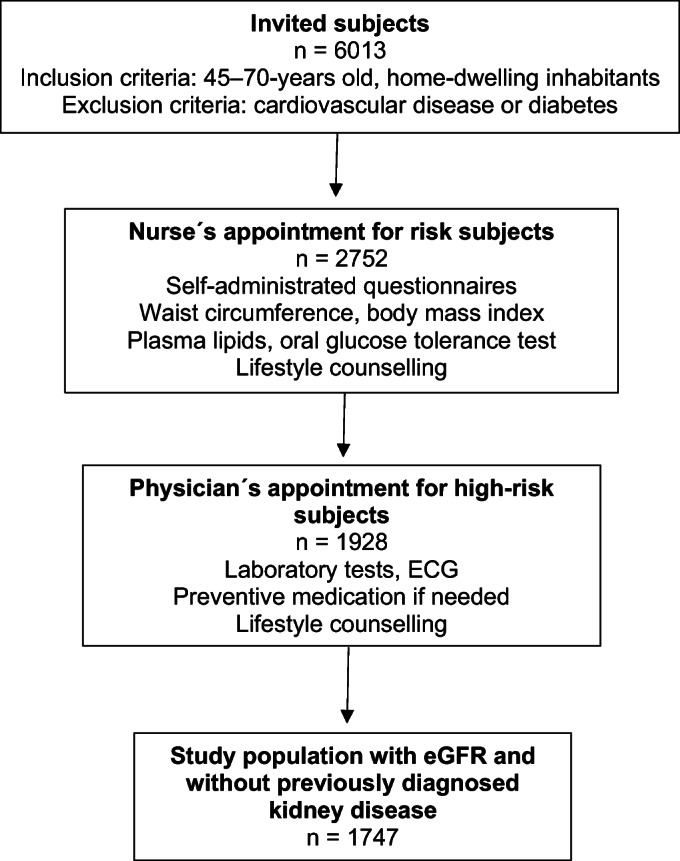
Formation of the study population. Abbreviations: SCORE, Systematic Coronary Risk Evaluation; ECG, electrocardiogram.

Harmonica Project (Harjavalta Risk Monitoring for Cardiovascular Disease) is a population-based screening and intervention program which was conducted in the semi-rural towns of Harjavalta and Kokemäki in Southwest Finland from autumn 2005 to autumn 2007.^13^ An invitation to the project, a cardiovascular risk factor survey, and type 2 diabetes risk assessment form (FINDRISC, Finnish Diabetes Risk Score, available from www.diabetes.fi/english)^14^ were mailed to all home-dwelling 45–70 years old residents (*n* = 6013). In the risk factor survey, the subjects were asked to measure waist circumference at the level of navel, report their latest measured BP (inclusion criteria ≥140/90 mmHg), and respond to the questions concerning use of antihypertensive medication, gestational diabetes and hypertension, and close relatives’ history of myocardial infarction or stroke. Subjects with at least one of the abovementioned CVD risk factors or ≥12 points in FINDRISC (≥15 in Kokemäki), were invited for further examinations. Response rate was 74%. In order to create a cohort in primary prevention, patients with previously diagnosed CVD or diabetes were excluded (*n*=274). Formation of the study population is illustrated in Fig. 1.

An appointment with a public health nurse was arranged for every eligible respondent (*n*=2752). BP was measured with a mercury sphygmomanometer in a sitting posture after at least 5-min rest with a suitable cuff placed on the arm. The mean of two BP readings taken at intervals of at least 2 min was used in the analysis. Pulse pressure was calculated as systolic BP − diastolic BP. Mean arterial pressure (MAP) was calculated as diastolic BP + 1/3 × (systolic BP − diastolic BP). If study subjects had no antihypertensive medication and the study nurse measured systolic BP ≥140 mmHg or diastolic BP ≥90 mmHg, subjects were taught to use an automatic validated BP monitor (Omron M4-1; Omron, Kyoto, Japan), which was lent to them for home BP monitoring to verify the hypertension diagnosis.

Height and weight were measured in a standing position without shoes and outer clothes. BMI was calculated as weight (kg) over height squared (m^2^). Waist circumference was measured at the level midway between lower rib margin and iliac crest.

Laboratory tests were performed after at least 12-h fasting. A 2-h oral glucose tolerance test (OGTT) was performed with a glucose load of 75 g dissolved in water. Glucose concentrations were measured from capillary whole blood samples using the HemoCue Glucose 201+ system (Ängerholm, Sweden) which converts values to plasma glucose concentrations. Plasma total cholesterol, triglycerides, and HDL cholesterol were measured enzymatically (Olympus AU604, Japan). LDL cholesterol was calculated by Friedewald’s formula.^15^

The participants filled in self-administrated questionnaires including smoking, alcohol consumption (Alcohol Use Disorders Identification Test, AUDIT^16^), leisure-time physical activity (LTPA), education, and health-related quality of life using the EuroQol instrument (EQ-5D)^17^.

Subjects with previously and newly detected hypertension, newly detected glucose disorders, obesity (BMI ≥30.0 kg/m^2^), metabolic syndrome, or the 10-year risk of cardiovascular death ≥5% calculated by the Systematic Coronary Risk Evaluation System (SCORE)^18^ were classified as high-risk subjects. Nurses gave individual lifestyle counseling for every subject.

The high-risk subjects (*n*=1928) were invited to a physician’s appointment within 2–4 months following the visit with a nurse. Fasting plasma lipids and glucose were retested, an ECG was taken, and laboratory tests were collected also to exclude secondary causes of dyslipidemia, hypertension, and glucose intolerance. Plasma creatinine was measured enzymatically (Olympus® AU640, Japan) and eGFR was calculated using the Chronic Kidney Disease Epidemiology Collaboration (CKD-EPI) formula^19^. Preventive antilipid and/or antihypertensive medication and low-dose aspirin was prescribed if the 10-year risk for a fatal cardiovascular event currently, or extrapolated to the age of 60 years, was ≥5% estimated by the SCORE system^18^. According to national guidelines at the time, antihypertensive medication was initiated if systolic BP was ≥160mmHg or diastolic BP ≥100 mmHg (in patients with hypertensive target organ damage or diabetes ≥140/90mmHg). The physician also gave personal lifestyle counseling at the appointment. The subjects with eGFR <60 ml/min/1.73 m^2^ were instructed to avoid nonsteroidal anti-inflammatory drugs.

For the analyses described here, we excluded participants with known CKD (polycystic kidney disease, chronic nephropathies, one single kidney), yielding an analytic cohort of 1747.

### Definitions

Subjects were categorized according to levels of kidney function based on the previously used classification eGFR <60, eGFR 60–74, eGFR 75–89, eGFR 90–104, and eGFR ≥105 ml/min/1,73m^2^ with the reference point at eGFR 90–104 ml/min/1,73 m^2^. When eGFR was characterized as a continuous variable, the reference point was set at 95 ml/min/1,73 m^2^.^1^

Type 2 diabetes was diagnosed with fasting glucose ≥7.0 mmol/l or 2-h postload glucose ≥12.2 mmol/l.^20^

Metabolic syndrome was diagnosed according to the criteria of the International Diabetes Federation (IDF) 2005 definition.^21^

LTPA was classified into three categories: high (LTPA for at least 30 min at a time for six or more times a week), moderate (LTPA for at least 30 min at a time for four to five times a week), and low (LTPA for at least 30 min at a time for a maximum of three times a week).

The Pharmacological Risk Assessment Online system (Pharao®) was used to identify potentially number of nephrotoxic drugs.^22^

### Morbidity and mortality data

Data on incident morbidity was obtained from the Care Register for Health Care of the National Institute of Health and Welfare containing data on inpatient care in Finnish hospitals. Data on mortality (all-causes and causes of death) was obtained from Statistics Finland. For each person, the date of the invitation to the Harmonica project was the start date of the observational period. For morbidity, follow-up ended on December 31st, 2018, or on the date of the first occurrence of incident CVD. For mortality, follow-up time ended on December 31st, 2019. Incident morbidity and causes of death were classified according to the International Statistical Classification of Diseases and Related Health Problems, 10th Revision (ICD-10). We categorized the endpoints of interest to ischemic heart disease (ICD-10 codes I20, I21, I25), heart failure (I50), cerebrovascular disease (I60, I61, I62, I65, I66), peripheral artery disease (PAD) I70, I71, I74), and atrial fibrillation or flutter (AF) (I48).

### Ethical Approval

The study protocol and consent forms were approved by the ethics committee of Satakunta hospital district. All participants provided written consent for the project and subsequent medical research. All tests were complimentary and voluntary for the participants.

### Statistical Analysis

Data are presented as means with standard deviation (SD) or as counts (*n*) with percentages (%). The unadjusted hypothesis of linearity was tested using the Cochran–Armitage test, analysis of variance, or logistic models with an appropriate contrast. The Kaplan–Meier method was applied to estimate the cumulative survival. Cox proportional hazards regression was used to estimate the adjusted hazard ratios (HR) and their 95% confidence intervals (CIs). Age, sex, current smoking, AUDIT score, new diabetes, total cholesterol, systolic BP level, and BMI were used as covariates in these models. The proportional-hazards assumption was evaluated by Schoenfeld residuals and log–log plots. A possible nonlinear relationship between mortality and eGFR was assessed by using 4-knot restricted cubic spline Cox regression models. The length of the distribution of knots was located at the 5th, 35th, 65th, and 95th percentiles. For restricted cubic splines, also known as natural splines, knot locations are based on Harrell’s recommended percentiles.^23^ The ratio of observed to expected number of deaths, the standardized mortality ratio (SMR) for all-cause deaths, was calculated using subject-years methods with 95% confidence intervals (CIs). The expected number of deaths was calculated on the basis of sex-, age-, and calendar-period-specific mortality rates in the Finnish population (Official Statistics of Finland). The normality of variables was evaluated graphically and by using the Shapiro–Wilk W test. Stata 17.0 (StataCorp LP, College Station, TX, USA) was used for the statistical analyses.

## RESULTS

### Characteristics of the Study Population

**Figure 2:**
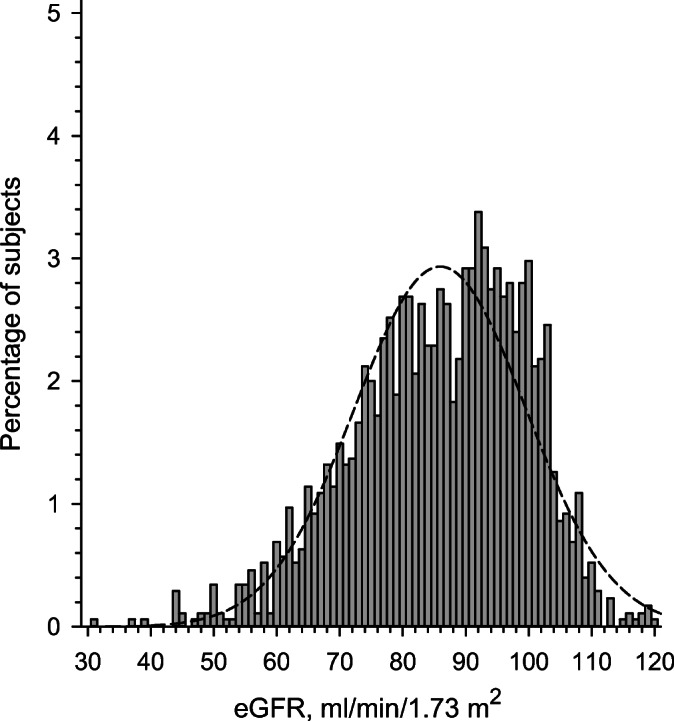
Distribution of estimated glomerular filtration rate (eGFR) in the study population. The dashed line indicates normal distribution.

At baseline, 1747 home-dwelling, 45–70-years old CVD risk subjects without manifested CVD, diabetes or kidney disease were evaluated. Their mean eGFR was 86 (SD 14) ml/min/1.73 m^2^ (range 31–120 ml/min). Figure 2 shows the distribution of eGFR values in this primary prevention cohort.

Subjects with eGFR <60 ml/min/1.73 m^2^ were more often females, and on average older and less educated than the other groups. Subjects with eGFR ≥105 ml/min were mostly males; had younger mean age, larger WC, and higher AUDIT score; smoked more often; and performed less LTPA than the other groups. Mean systolic BP and pulse pressure values, and usage of antihypertensive medication rose linearly with declining eGFR. Subjects with eGFR <60 ml/min had lower diastolic BP level, higher 2-h glucose concentration, and higher prevalence of metabolic syndrome, and they used more often lipid lowering medication compared to the other groups (Table 1).

**Table 1:** Baseline Characteristics of the 1747 Subjects According to Categories of estimated Glomerular Filtration Rate (eGFR)

	eGFR category (ml/min/1.73 m^2^)					*P* for linearity
	<60 *N*=58	60–74 *N*=296	75–89 *N*=603	90–104 *N*=693	≥105 *N*=97	
Age, years, mean (SD)	64 (6)	61 (6)	60 (6)	57 (6)	51 (5)	<0.001
Females, *n* (%)	48 (83)	204 (69)	325 (54)	303 (44)	36 (37)	<0.001
Education years, mean (SD)	9.8 (3.0)	10.0 (2.8)	10.1 (2.7)	10.3 (2.6)	11.3 (2.5)	<0.001
Body mass index, kg/m^2^, mean (SD)	29.9 (5.4)	30.0 (5.0)	29.9 (4.9)	30.0 (5.0)	30.6 (5.8)	0.55
Waist circumference, cm, mean (SD)						
Women	96 (13)	95 (13)	95 (13)	96 (13)	103 (15)	0.22
Men	98 (9)	102 (9)	103 (10)	103 (11)	105 (13)	0.034
Current smoking, *n* (%)	8 (14)	28 (9)	88 (15)	145 (21)	39 (40)	<0.001
AUDIT score, mean (SD)	2.3 (3.5)	3.7 (4.0)	4.3 (4.4)	5.8 (5.5)	8.0 (7.3)	<0.001
LTPA level, *n* (%)						<0.001
Low	13 (23)	42 (15)	101(17)	165 (25)	29 (31)	
Moderate	26 (46)	137 (48)	283 (49)	338 (50)	48 (51)	
High	17 (30)	104 (37)	197 (34)	168 (25)	18 (19)	
Blood pressure, mmHg, mean (SD)						
Systolic	149 (16)	149 (18)	148 (18)	147 (18)	143 (18)	0.010
Diastolic	85 (9)	88 (8)	88 (8)	88 (9)	88 (8)	0.049
Pulse pressure	63 (15)	61 (14)	60 (14)	59 (14)	54 (13)	<0.001
Mean arterial pressure	107 (9)	108 (10)	108 (10)	107 (11)	107 (11)	0.72
Plasma lipids, mmol/l, mean (SD)						
Total cholesterol	5.45 (1.00)	5.29 (1.00)	5.29 (0.98)	5.26 (0.97)	5.22 (1.02)	0.12
HDL cholesterol	1.54 (0.41)	1.46(0.41)	1.48 (0.44)	1.48 (0.46)	1.42(0.44)	0.35
LDL cholesterol	3.27 (0.94)	3.24 (0.86)	3.19 (0.86)	3.21 (0.85)	3.15 (0.90)	0.41
Triglycerides	1.48 (0.70)	1.41 (0.67)	1.43 (1.06)	1.38 (0.73)	1.49 (0.85)	0.52
Plasma glucose, mmol/l, mean (SD)						
Fasting	5.69 (0.91)	5.61 (0.78)	5.67 (0.98)	5.72 (1.27)	5.96 (1.50)	0.096
2-h glucose	8.24 (2.48)	8.07 (2.37)	7.88 (2.46)	7.55 (2.44)	7.29 (2.31)	<0.001
New type 2 diabetes, *n* (%)	8 (14)	27 (9)	54 (9)	74 (11)	10 (10)	0.86
Metabolic syndrome, *n* (%)	45 (78)	189 (64)	370 (61)	411 (59)	69 (71)	0.043
EQ-5D score, mean (SD)	0.759 (0.230)	0.825 (0.196)	0.823 (0.162)	0.815 (0.173)	0.801 (0.185)	0.31
Regular medication, *n* (%)						
Lipid-lowering	14 (24)	58 (20)	118 (20)	89 (13)	11 (11)	<0.001
Antihypertensive	39 (67)	160 (54)	275 (46)	289 (42)	29 (30)	<0.001
Nephrotoxic drugs	6 (10)	16 (5)	29 (5)	33 (5)	3 (3)	0.055

**Abbreviations:** *AUDIT* Alcohol Use Disorders Identification Test, *LTPA* leisure-time physical activity, *HDL* high-density lipoprotein, *LDL* low-density lipoprotein

### Morbidity

In the whole cohort, a total of 20,818 person-years was followed up for morbidity. In comparison to eGFR category 90–104 ml/min/1.73 m^2^ at baseline, subjects with eGFR 60–74 ml/min had a decreased risk of heart failure, whereas subjects with eGFR ≥105 ml/min had an increased risk of incident PAD, AF, and any first endpoint of interest when adjustments were made for age, sex, current smoking, AUDIT score, new diabetes, total cholesterol, systolic BP level, and BMI (Table 2).

**Table 2:** The Number of and Adjusted Hazard Ratios (HR) with 95% Confidence Intervals (CIs) for Incident Endpoints According to Categories of Estimated Glomerular Filtration Rate (eGFR). Adjustments Made for Age, Sex, Current Smoking, AUDIT Score, New Diabetes, Total Cholesterol, Systolic Blood Pressure Levels, and Body Mass Index

Endpoint (ICD-10 codes)	eGFR category (ml/min/1.73 m^2^)				
	<60 *N*=58	60–74 *N*=296	75–89 *N*=603	90–104 *N*=693	≥105 *N*=97
Heart failure (I50)					
Number (%)	5 (9)	9 (3)	27 (4)	33 (5)	4 (4)
Adjusted HR (95% CI)	1.01 (0.38 to 2.70)	0.45 (0.21 to 0.96)	0.67 (0.39 to 1.13)	1.00 Reference	1.98 (0.66 to 5.92)
Ischemic heart disease (I20, I21, I25)					
Number (%)	11 (19)	26 (9)	66 (11)	63 (9)	6 (6)
Adjusted HR (95% CI)	1.47 (0.75 to 2.87)	0.79 (0.49 to 1.27)	0.98 (0.69 to 1.40)	1.00 Reference	1.12 (0.47 to 2.66)
Cerebrovascular disease (I60, I61, I62, I65, I66)					
Number (%)	5 (9)	18 (6)	47 (8)	41 (6)	6 (6)
Adjusted HR (95% CI)	1.00 (0.38 to 2.60)	0.89 (0.50 to 1.58)	1.17 (0.76 to 1.80)	1.00 Reference	1.56 (0.64 to 3.79)
Peripheral artery disease (I70, I71, I74)					
Number (%)	4 (7)	8 (3)	20 (3)	19 (3)	6 (6)
Adjusted HR (95% CI)	2.21 (0.70 to 6.94)	1.16 (0.49 to 2.76)	1.21 (0.63 to 2.30)	1.00 Reference	2.62 (1.00 to 6.94)
Atrial fibrillation or flutter (I48)					
Number (%)	5 (9)	36 (12)	75 (12)	97 (14)	17 (18)
Adjusted HR (95% CI)	0.48 (0.19 to 1.20)	0.75 (0.50 to 1.11)	0.76 (0.55 to 1.03)	1.00 Reference	2.10 (1.21 to 3.65)
Any first endpoint					
Number (%)	21 (36)	68 (23)	161 (27)	177 (26)	27 (28)
Adjusted HR (95% CI)	1.12 (0.70 to 1.79)	0.80 (0.60 to 1.07)	0.93 (0.75 to 1.16)	1.00 Reference	1.69 (1.11 to 2.59)

### Cumulative All-Cause Mortality

For mortality, a total of 22,347 person-years was followed up in the whole cohort, and 230 deaths occurred. Crude cumulative all-cause mortality in the categories of eGFR over 14 years of follow-up was as follows: 24.1% (95% CI: 15.1 to 37.3) in eGFR <60 ml/min/1.73 m^2^, 14.8% (95% CI: 11.0 to 20.0) in eGFR 60–74 ml/min, 12.4% (95% CI: 10.0 to 18.7) in eGFR 75–89 ml/min, 13.5% (95% CI: 11.1 to 16.5) in eGFR 90–104 ml/min, and 24.0% (95% CI: 13.3 to 40.8) in eGFR ≥105 ml/min.

### Risk of Death

**Figure 3:**
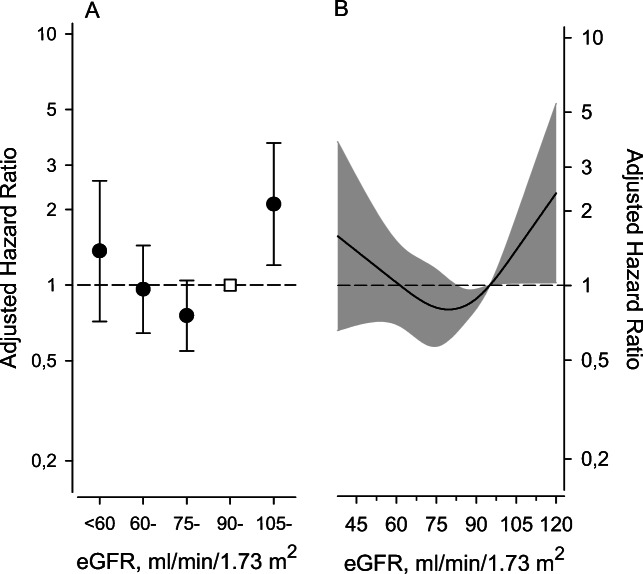
Adjusted hazard ratios for all-cause mortality according to estimated glomerular filtration rate (eGFR) categories with 90–104 ml/min/1.73 m^2^ set as the reference (panel A). Panel B shows the adjusted hazard ratios according to continuous eGFR values with eGFR 95 ml/min/1.73 m^2^ set as the reference and derived from a 4-knot restricted cubic spline. Cox proportional hazards regression model. Whiskers and the gray area represent the 95% confidence intervals. Adjustments were made for age, sex, current smoking, AUDIT score, new diabetes, total cholesterol, systolic blood pressure levels, and body mass index.

Compared with eGFR category 90–104 ml/min/1.73 m^2^, adjusted hazard ratio (HR) for all-cause mortality was 2.15 (95% CI: 1.24 to 3.73) for eGFR category ≥105 ml/min, 1.33 (95% CI: 0.70 to 2.51) for category <60 ml/min, 0.95 (95% CI: 0.63 to 1.42) for eGFR 60–74 ml/min, and 0.73 (95% CI: 0.53 to 1.01) for eGFR 75–89 ml/min, *P* = 0.003 (quadratic contrast) (Fig. 3A). For eGFR category ≥105 ml/min, crude HR for all-cause mortality was 1.38 (95% 0.82 to 2.31), and crude incidence rate 14 (18 to 22) per 1000 person years.

### Standardized Mortality Ratio

**Figure 4:**
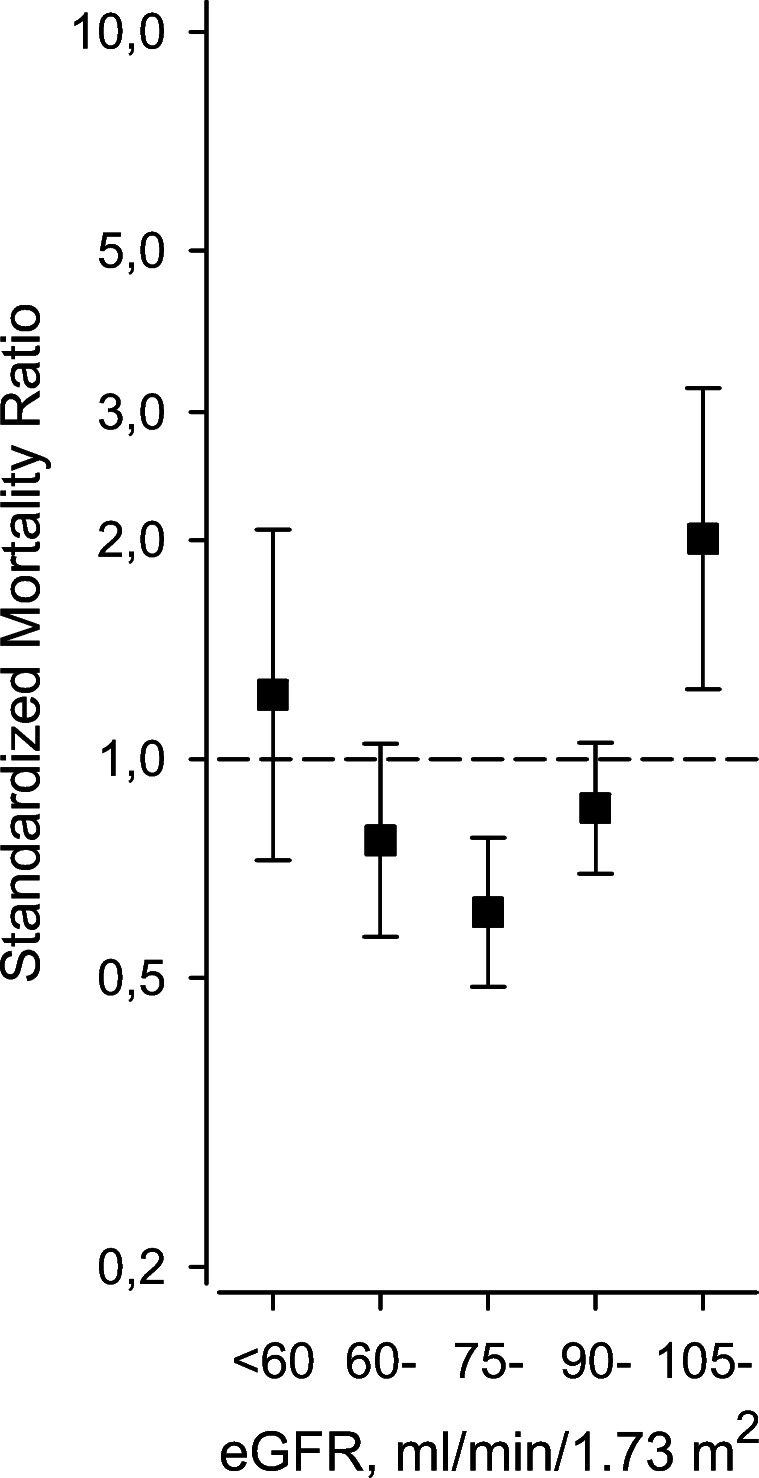
Standardized mortality ratio according to the categories of estimated glomerular filtration rate (eGFR).

The eGFR category 75–89 ml/min/1.73 m^2^ was associated with a decreased SMR of 0.62 (95% CI 0.49 to 0.78), whereas the highest eGFR category ≥105 ml/min/1.73 m^2^ was associated with increased SMR of 2.01 (95% CI 1.5 to 3.23), *P* <0.001 (quadratic contrast) (Fig. 4).

### Causes of Death

Of the 230 deaths occurred, 14 (6.1%) were among those with eGFR <60 ml/min/1.73 m^2^. There were 41 (17.8%) deaths in eGFR category 60–74 ml/min, 69 (30.0%) in eGFR 75–89 ml/min, 89 (38.7%) in eGFR 90–104 ml/min, and 17 (7.4%) in eGFR category ≥105 ml/min. The most prevalent cause of death was cancer (42.2% of all deaths), followed by CVD (29.6% of all deaths). The causes of death according to categories of eGFR are presented in Supplementary Appendix Table.

## DISCUSSION

In this prospective cohort study of apparently healthy cardiovascular risk subjects, eGFR category ≥105 ml/min/1.73 m^2^ calculated by the CKD-EPI equation was associated with a two-fold risk of all-cause mortality when compared to eGFR category 90–104 ml/min over the 14-year follow-up. Moreover, subjects with eGFR ≥105 ml/min at baseline had an increased risk of incident PAD and AF in comparison to eGFR category 90–104 ml/min. These results remained statistically significant after adjustment with several cardiovascular and lifestyle-related risk factors. The highest eGFR category ≥105 ml/min was also associated with a two-fold increased mortality rate, and eGFR category 75–89 ml/min with a 38% decreased mortality rate compared to the Finnish general population.

In the meta-analyses of 11 general-population studies with over 90,000 participants (mean eGFR 85 ml/min/1.73 m^2^), a reverse J-shaped association between all-cause mortality and eGFR calculated by the CKD-EPI equation was observed.^1^ As in our study, increased mortality was detected among those with eGFR ≥105 ml/min. However, when using the cystatin C-based eGFR and eGFR based on combined measurements of creatinine and cystatin C, the association between eGFR ≥105 ml/min and increased mortality risk was diminished.^1^ The difference was speculated to reflect confounding by non-GFR determinants of creatinine and cystatin C.^1^ These include muscle mass, diet, and physical activity for creatinine level, and obesity, inflammation, and diabetes for cystatin C level.^24–27^ Our study population was apparently healthy and had good health-related quality of life throughout the eGFR levels, and only two were underweighted (BMI 17.9 kg/m^2^). Surprisingly, those with eGFR ≥105 ml/min had the highest mortality risk even though they were younger and had less traditional cardiovascular risk factors than subjects in the other eGFR categories. Still, it is noteworthy that subjects with the highest eGFR level had more unfavorable lifestyle habits, abdominal obesity, and metabolic syndrome. Thus, eGFR ≥105 ml/min calculated by the CKD-EPI equation seems to be a surrogate for elevated mortality risk among apparently healthy cardiovascular risk subjects.

Compared to the mortality rate in the Finnish general population over the same period, SMR was decreased among the subjects in the eGFR category 75–89 ml/min but increased in the highest eGFR category. We are not aware of other studies investigating the association of eGFR levels with SMR. However, in the meta-analysis by Shlipak et al., a significantly lowered risk of death at eGFR level 80–90 ml/min was also observed.^1^

To our knowledge, the association of renal hyperfiltration and increased risk of PAD or AF has not been reported earlier. We could take into account many confounding risk factors for these conditions but still, subjects with eGFR ≥105 ml/min had a two-fold risk for PAD and AF. One may speculate that plasma volume overload predisposes to structural changes in heart and subsequent arrythmia. However, eGFR ≥105 ml/min was not associated with incident heart failure, ischemic heart disease, or cerebrovascular events in our study population. Instead, eGFR level 60–74 ml/min at baseline seemed to protect against heart failure. We cannot explain the pathophysiological mechanisms behind these observations with our data. However, renal hyperfiltration has been related to low-grade systemic inflammation and oxidative stress, left ventricular hypertrophy, and an increased incidence of coronary artery calcification.^28–31^ Thus, renal hyperfiltration may play a role in the genesis of AF and different localizations or early manifestations of atherosclerosis. The observed impact of eGFR ≥105 ml/min on AF risk suggests that opportunistic AF screening of individuals with renal hyperfiltration by pulse taking may be warranted.

According to our results, more patients treated in primary care are at high risk for mortality because of higher-than-normal eGFR, rather than lower-than-normal eGFR. The prevalence of CKD defined as eGFR <60 ml/min/1.73 m^2^ was 3% but was not associated with elevated risk for all-cause mortality or cardiovascular morbidity. It is noteworthy that none of the study participants had eGFR <30 ml/min at baseline. Six percent of the participants had eGFR ≥105 ml/min thus belonging to the eGFR category with the highest risk for AF, PAD, and mortality. The screening method used took in account the conventional risk factors for CVD and type 2 diabetes, and excluded individuals with manifested CVD, diabetes, or renal disease. Clearly, studies are needed to quantify benefits and harms of screening, to evaluate screening measures (e.g., eGFR equations, cystatin C, albuminuria) and target groups (e.g., older age, obesity, CVD).

There are limitations in our study. Despite a long follow-up period, the number of deaths was quite low both in the eGFR category of <60 ml/min and ≥105 ml/min at baseline. This may lower the power of the study. Moreover, the increased risk of incident PAD and AF among subjects with eGFR ≥105 ml/min needs to be confirmed in larger study populations. We did not measure urinary albumin excretion or hematuria, and GFR was estimated only on one occasion at baseline. However, the risk associations of eGFR and albuminuria categories appear to be largely independent of one another.^5^ Our study population is a representative sample of 45–70-year-old patients typically treated in primary care, but all of them are of Caucasian origin and our results may not be generalized to other age groups or ethnic groups. Nor can the results be generalized to subjects without CVD risk factors. The baseline clinical measurements were made by trained medical staff, and data on mortality were obtained from a national register with high validity.^32^ Currently, there is no consensus whether renal hyperfiltration should be defined as a cut-off value of eGFR or as a value of eGFR over the 95th percentile. However, dichotomization of the continuous variables makes clinical decisions easier. In a recent study with a 35-year follow-up conducted among Finnish male subjects, the eGFR value of 97 ml/min/1.73 m^2^ served as an optimal cut-off for renal hyperfiltration corresponding to the hazard of all-cause mortality with the highest accuracy.^33^

## CONCLUSIONS

Renal hyperfiltration defined as eGFR ≥105 ml/min/1.73 m^2^ is a frequent and important finding in patients commonly treated in primary care. Renal hyperfiltration is associated with increased risk of all-cause mortality, PAD, and AF. These patients should be followed closely for timely interventions.

## Supplementary Information

ESM 1 (DOCX 14 kb)

## Data Availability

The datasets during and/or analyzed during the current study available from the corresponding author on reasonable request.
