# Supplementary material for: The Relationship of Kidney Function, Cardiovascular Morbidity, and All-Cause Mortality: a Prospective Primary Care Cohort Study
Source: J Gen Intern Med. 2022 Dec 21;38(8):1834–42. doi: 10.1007/s11606-022-07885-8 (PMC10271946; doi:10.1007/s11606-022-07885-8)
Supplement: Supplementary file 1 — (DOCX 14 kb) [file 11606_2022_7885_MOESM1_ESM.docx]

**Supplementary Appendix Table.** **Causes of death according to categories of estimate glomerular filtration rate (eGFR)**

|  | eGFR category (ml/min/1.73 m^2^) | | | | |
| --- | --- | --- | --- | --- | --- |
| Causes of death  (ICD-10 codes) | <60  (N=14)  n (%) | 60–74  (N=41)  n (%) | 75–89  (N=69)  n (%) | 90–105  (N=89)  n (%) | ≥105 (N=17)  n (%) |
| Malignant neoplasms  (C00-D48) | 4 (29) | 21 (51) | 21 (30) | 46 (52) | 5 (29) |
| Diseases of the nervous system (G00-G99) | 1 (7) | 3 (7) | 8 (12) | 2 (2) | 1 (6) |
| Diseases of the circulatory system (I00-I99) | 4 (29) | 11 (27) | 22 (32) | 27 (30) | 4 (24) |
| Diseases of the respiratory system (J00-J99) | 1 (7) | 0 (0) | 5 (7) | 1 (1) | 1 (6) |
| Diseases of the digestive system (K00-K93) | 1 (7) | 1 (2) | 1 (1) | 5 (6) | 4 (24) |
| External causes  (V01-Y98) | 0 (0) | 5 (12) | 10 (14) | 7 (8) | 1 (6) |
| Other causes | 3 (21) | 0 (0) | 2 (3) | 1 (1) | 1 (6) |
